# The association between nutritional risk and survival time among patients with pancreatic cancer following pancreaticoduodenectomy: a retrospective cohort study

**DOI:** 10.3389/fonc.2025.1539215

**Published:** 2025-07-01

**Authors:** Qiuju Tian, Jing Su, Leying Chen, Min Zhang, Beiwen Wu, Baiyong Shen

**Affiliations:** ^1^ Gastroenterology Department, Ruijin Hospital, Affiliated with Shanghai Jiao Tong University School of Medicine, Shanghai, China; ^2^ Nursing Department, Ruijin Hospital, Affiliated with Shanghai Jiao Tong University School of Medicine, Shanghai, China; ^3^ Pancreatic Surgery Department, Ruijin Hospital, Affiliated with Shanghai Jiao Tong University School of Medicine, Shanghai, China; ^4^ Party Committee Office, Ruijin Hospital, Affiliated with Shanghai Jiao Tong University School of Medicine, Shanghai, China

**Keywords:** nutritional risk, screening, pancreatic cancer, pancreaticoduodenectomy, survival

## Abstract

**Background:**

Nutritional problems are common in patients with pancreatic cancer. However, the relationship between nutritional risk screening and the survival of patients after pancreaticoduodenectomy remains inconclusive. This study aimed to examine the association between preoperative nutritional risk and survival time among adult Chinese patients with pancreatic cancer after pancreaticoduodenectomy.

**Methods:**

This study was conducted at Ruijin Hospital, affiliated with Shanghai Jiao Tong University School of Medicine in China. Patients aged 18 years or more who received pancreaticoduodenectomy for pancreatic cancer in our center between December 2019 and June 2022 from the follow-up database were included in the study. We retrospectively collected data on the demographics, disease, treatment, nutritional risk score, and survival time of the patients with pancreatic cancer. A Cox regression model was used to analyze the association between nutritional risk and survival time in different covariate models.

**Results:**

A total of 656 patients were included in the study, and the median survival time was 24.0 months (95% CI:21.6-26.3). In total, 29.1% of patients had nutritional risk on admission. At the end of the follow-up, a total of 364 (55.5%) patients had died. The overall 1-, 2-, and 3-year survival rate of the 656 patients with pancreatic cancer after pancreaticoduodenectomy was 72.7%, 49.8%, and 34.4%, respectively. In the Cox regression model adjusted for age, education level, carbohydrate antigen 199 levels, neutrophil-lymphocyte ratio, tumor diameter, lymph node metastasis, distant organ metastasis, differentiation, nerve invasion, surgical margins, surgical time, intraoperative blood loss, postoperative complications, and chemotherapy, patients with nutritional risk score greater than 3 had a lower survival time compared with those without nutritional risk (HR = 1.33, 95% CI:1.06–1.67; P = 0.015).

**Conclusions:**

Preoperative nutritional risk has a detrimental impact on survival in patients with pancreatic cancer who undergo pancreaticoduodenectomy, and this relationship is stable. Nursing staff should screen early for nutritional risk using the Nutritional Risk Screening-2002 tool in patients with pancreatic cancer at diagnosis and, in conjunction with their doctors, develop and implement a timely nutritional treatment plan for those at risk to improve the poor survival time.

## Introduction

Pancreatic cancer is a malignancy with a poor prognosis and high mortality. The development of early diagnostic techniques has increased the chances of success of surgical resection (1–3). Pancreaticoduodenectomy is a classic surgical approach to treat tumors in the head or body of the pancreas. With the refinement of surgical techniques, combined with the improvement of adjuvant and neoadjuvant therapy, prolonged survival in patients with resectable pancreatic cancer has been achieved (4). The 5-year relative survival rate for pancreatic cancer has increased from 3% for diagnoses during the mid-1970s to 13% during 2013–2019 (5). Factors associated with pancreatic cancer survivor survival time in previous studies include age (6), carbohydrate antigen 199 levels, neutrophil-lymphocyte ratio (NLR), tumor diameter, lymph node metastasis, distant organ metastasis, resection margin status, vascular resection, adjuvant chemotherapy, and differentiation (7–10). However, the results of different studies vary considerably as to whether nutritional indicators have an impact on the survival of patients after pancreatic cancer surgery.

Nutritional problems, which are common in patients with advanced pancreatic cancer, not only affect disease progression but also increase mortality. Nutritional Risk Screening-2002 (NRS-2002) is an easily applied and reproducible tool to predict the nutritional risk for patients in the hospital, which has been used widely to identify the risk for surgical complications and survival outcome (11, 12). The European Society for Clinical Nutrition and Metabolism (ESPEN) also recommends the application of NRS-2002 in patients undergoing surgery and patients with cancer (13). Heckler et al. found that nutritional risk defined by NRS-2002 was not associated with worse survival in patients with resected pancreatic ductal adenocarcinoma (14), while Park found that the NRS-2002 score was associated with overall survival in patients with advanced pancreatic cancer (15). Thus, the relationship between the NRS-2002 and the survival of pancreatic cancer patients after pancreaticoduodenectomy needs more studies.

In the present study, we aimed to examine the associations between baseline nutritional risk and survival time among adult Chinese patients with pancreatic cancer after pancreaticoduodenectomy. We hope that the identification of nutritional risk indicators will contribute to the development and implementation of preoperative nutrition improvement programs and ultimately improve the survival of patients with pancreatic cancer after pancreaticoduodenectomy.

## Methods and materials

### Study population

This was a retrospective study. We selected patients aged 18 years or more who received pancreaticoduodenectomy treatment for pancreatic cancer at Ruijin Hospital, affiliated with Shanghai Jiao Tong University School of Medicine, between December 2019 and June 2022 from the follow-up database. Patients without a clinical data record were excluded. The study protocol was approved by the institutional review board at the authors’ affiliated hospital [Ethical Review Approval No. 293 (2023)].

### Data collection

We retrospectively collected patients’ clinical data from medical records and the follow-up database. The follow-up database contains the patient’s hospitalization number, age, gender, height, body weight, body mass index (BMI), comorbidities, postoperative complications, survival information, neoadjuvant therapy, and postoperative chemotherapy information. All the patients were followed up at regular intervals via telephone for routine clinical care every 3 months during the first year in our center after treatment with pancreaticoduodenectomy. The follow‐up ended when the patient died or contact was lost. Our most recent follow‐up was conducted in August 2023, and patient survival data were censored. Every patient in our study received at least 1 year of follow‐up after discharge from the hospital. We focused on the patients’ overall survival results, which were defined as the time from treatment to death due to any cause.

We then logged on to the inpatient electronic medical record system and used the patient’s hospitalization number to find out the patient’s marital status, time of entry and exit from the hospital, education level, ethnicity, smoking status, alcohol consumption, amount of weight lost and time of loss, any reduction in diet in the last week, Barthel’s index, carbohydrate antigen 199 levels on admission, neutrophil value on admission, lymphocyte value on admission, surgical margins, lymph node metastasis, distant organ metastasis, nerve invasion, maximum diameter of the tumor, and the degree of tumor differentiation. We determined the cut-off values for NLR, tumor diameter, operation time, and intraoperative blood loss based on the ROC curve, and divided these continuous variables into binary variables based on the cut-off values.

The assessments of nutritional risk score using the NRS-2002 are available from the electronic medical record system. The NRS-2002 assesses nutritional risk using the medical record data on weight loss, BMI, food intake, disease severity, and age. Patient scores in the NRS-2002 are calculated as the score of impaired nutrition status and disease severity. If the patient’s age is ≥70 years, one point is added to the total score. Patients with an NRS-2002 score <3 were classified as “without nutritional risk” and those with a score of 3 or more were classified as “with nutritional risk”.

If a patient is identified as having nutritional risk via NRS 2002, the general approach is to recommend oral nutritional supplements (ONSs) if they are able to tolerate oral intake. For patients diagnosed with malnutrition and unable to meet their nutritional requirements orally, a consultation with the nutrition support team is requested, potentially leading to the initiation of total parenteral nutrition (TPN) if necessary. All of the patients in our center received nutritional support during the postoperative hospitalization after their pancreaticoduodenectomy, but the access to and initiation of preoperative nutritional interventions can depend significantly on the individual clinician’s assessment and prioritization. Furthermore, a preoperative nutritional intervention is recommended to begin 7 to 14 days before surgery (16). However, in practice, the patients received a shorter duration of nutritional support preoperatively. So, in our study, if the patients were prescribed enteral nutrition preparations, intravenous glucose, amino acids, or fat emulsion before surgery, they were considered as having received a nutritional intervention.

### Statistical analysis

The statistical software SPSS 20.0 was used in the statistical analysis. If the continuous variables were normally distributed, they are presented as mean ± standard deviation. Otherwise, they are presented as median (interquartile range). Categorical variables are presented as percentages. The survival rate and median overall survival time were calculated using the Kaplan–Meier method; then, survival curves were drawn. The log-rank test was used to compare the differences among the groups for the univariate analysis. Factors with statistical significance in the univariate analysis were included in the Cox regression model for the multivariate analysis. In addition, as nutritional support was not a standardized intervention and was also administered for an insufficient period, we used this nutritional support variable as a stratification variable in the Cox regression model to analyze the relationship between nutritional risk and the survival outcomes. A two‐sided p-value of less than 0.05 was considered to be statistically significant.

## Results

### General information

A total of 656 patients who underwent a pancreaticoduodenectomy were included in the present study, with a median age of 65 (58, 70) years old. In total, 29.1% of patients were at nutritional risk on admission. The median overall survival time was 24.0 months [95% confidence interval (CI), 21.6–26.3]. At the end of the follow-up, a total of 364 (55.5%) patients had died. The overall 1-, 2-, and 3-year survival rate of the 656 patients with pancreatic cancer after pancreaticoduodenectomy was 72.7%, 49.8%, and 34.4%, respectively (**Table 1**).

**Table 1:** Characteristics of the participants included in the study.

Variable	N (%)
Gender, no. (%)	
Male	395 (60.2)
Female	261 (39.8)
Age (years), no. (%)	
< 60	206 (31.4)
>=60	450 (68.6)
Education level, no. (%)	
Junior school or below	355 (54.1)
High school or above	301(45.9)
Marital status, no. (%)	
Married	646 (98.5)
Single	10 (1.5)
Smoking status, no. (%)	
Current smoker	174 (26.5)
Non-current smoker	482 (73.5)
Drinker, no. (%)	
Drinker	115 (17.5)
Non-drinker	541 (82.5)
Carbohydrate antigen 199 levels, no. (%)	
<= 305	419 (63.9)
> 305	237 (36.1)
Neutrophil-lymphocyte ratio, no. (%)	
< 2.93	353 (53.8)
>= 2.93	303 (46.2)
Tumor diameter (cm), no. (%)	
< 2.65	257 (39.2)
>= 2.65	399 (60.8)
Lymph node metastasis, no. (%)	
No	303 (46.2)
Yes	353 (53.8)
Distant organ metastasis, no. (%)	
No	624 (95.1)
Yes	32 (4.9)
Differentiation, no. (%)	
High	8 (1.2)
High-moderate and moderate	390 (59.5)
Moderate-poor and poor	256 (39.0)
Other	2 (0.3)
Nerve invasion, no. (%)	
No	35 (5.3)
Yes	621 (94.7)
Surgical margins, no. (%)	
R0	647 (98.6)
R1	9 (1.4)
Surgical time (minutes), no. (%)	
< 282.5	218 (33.2)
>= 282.5	438 (66.8)
Intraoperative blood loss (ml), no. (%)	
< 450	505 (77.0)
>= 450	151 (23.0)
Postoperative complications, no. (%)	
No	410 (62.5)
Yes	246 (37.5)
Adjuvant chemotherapy, no. (%)	
No	208 (31.7)
Yes	448 (68.3)
NRS-2002 score, no. (%)	
< 3	465 (70.9)
>= 3	191 (29.1)
Nutritional support before surgery, no. (%)	
No	284 (43.3)
Yes	372 (56.7)

### Analysis of prognostic factors for pancreatic cancer patient survival after pancreaticoduodenectomy

The univariate analysis showed that age, education level, carbohydrate antigen 199 levels, NLR, tumor diameter, lymph node metastasis, distant organ metastasis, tumor tissue differentiation, nerve invasion, surgical margins, postoperative complications, operative time, intraoperative blood loss, chemotherapy, and NRS-2002 score were the relevant factors affecting the prognosis of patients with pancreatic cancer after pancreaticoduodenectomy (*P* < 0.05) (**Table 2**).

**Table 2:** Univariate analysis of prognostic factors for the survival of patients with pancreatic cancer.

Variable	Median survival (95% confidence interval)	χ^2^	*P*
Gender		0.072	0.789
Male	24.73 (21.08–28.39)		
Female	23.63 (20.19–27.08)		
Age (years)		9.317	0.002
< 60	30.23 (23.93–36.53)		
>= 60	22.07 (19.39–24.73)		
Education level		6.303	0.012
Junior school or below	21.63 (19.18–24.08)		
High school or above	27.23 (23.69–30.78)		
Smoking status		0.001	0.976
Current smoker	23.63 (19.61–27.65)		
Non-current smoker	23.97 (21.15–26.79)		
Drinker		0.033	0.855
Drinker	23.37 (20.14–26.60)		
Non-drinker	24.73 (21.14–28.33)		
Carbohydrate antigen 199 levels		17.138	<0.001
<= 305	27.23 (24.03–30.43)		
> 305	18.33 (14.53–22.14)		
Neutrophil-lymphocyte ratio		20.63	<0.001
< 2.93	30.23 (26.12–34.35)		
>= 2.93	19.40 (16.12–22.67)		
Tumor diameter		8.76	0.003
< 2.65	29.20 (22.88–35.53)		
>= 2.65	21.97 (18.90–25.03)		
Lymph node metastasis		31.36	< 0.001
No	32.10 (28.91–35.29)		
Yes	19.40 (16.43–22.38)		
Distant organ metastasis		32.678	< 0.001
No	25.43 (22.21–28.66)		
Yes	9.43 (5.09–13.78)		
Differentiation		26.919	< 0.001
High	34.87 (18.38–38.05)		
High-moderate and moderate	30.10 (26.66–33.54)		
Moderate-poor and poor	16.47 (12.85–20.09)		
Other	25.23 (25.23–25.23)		
Nerve invasion		4.036	0.045
No	30.10 (28.90–31.31)		
Yes	23.37 (20.98–25.75)		
Surgical margins		7.909	0.005
R0	24.73 (21.98–27.49)		
R1	9.97 (3.83–16.10)		
Surgical time		13.970	< 0.001
< 282.5 minutes	31.63 (26.28–36.98)		
>= 282.5 minutes	22.03 (19.32–24.24)		
Intraoperative blood loss		12.55	< 0.001
< 450 ml	26.70 (23.08–30.33)		
>= 450 ml	17.87 (13.43–22.30)		
Postoperative complications		8.917	0.003
No	26.70 (22.83–30.57)		
Yes	21.63 (18.45–24.82)		
Chemotherapy		17.58	< 0.001
No	18.03 (13.51–22.56)		
Yes	26.93 (23.50–30.37)		
NRS2002 value		12.381	< 0.001
< 3	26.50 (22.73–30.27)		
>= 3	20.13 (15.52–24.74)		
Nutritional support before surgery		7.729	0.007
No	28.63 (23.16–34.11)		
Yes	22.03 (19.11–24.96)		

NRS-2002, Nutritional Risk Screening-2002.

Significant factors in the univariate analysis were included in the Cox regression model for multivariate analysis, with nutritional support variable as a stratification variable. The results showed that carbohydrate antigen 199 levels, NLR, tumor diameter, lymph node metastasis, distant organ metastasis, tumor tissue differentiation, surgical margins, operative time, postoperative complications, chemotherapy, and NRS-2002 score were independent prognostic factors in patients with pancreatic cancer after pancreaticoduodenectomy (*P* < 0.05) (**Table 3**).

**Table 3:** Multivariate analysis of prognostic factors for the survival of patients with pancreatic cancer.

Variable	Hazard ratio	*95% confidence interval*	*P*
Age (years)	1.21	(0.94–1.55)	0.140
< 60 (ref.)			
>=60			
Education level	0.87	(0.70–1.08)	0.200
Junior school or below (ref.)			
High school or above			
Carbohydrate antigen 199 levels	1.38	(1.11–1.72)	0.004
<=305 (ref.)			
>305			
Neutrophil-lymphocyte ratio	1.44	(1.16–1.79)	0.001
< 2.93 (ref.)			
>= 2.93			
Tumor diameter	1.40	(1.11–1.75)	0.004
< 2.65 (ref.)			
>= 2.65			
Lymph node metastasis	1.68	(1.35–2.10)	< 0.001
No (ref.)			
Yes			
Distant organ metastasis	1.93	(1.27–2.92)	0.002
No (ref.)			
Yes			
Differentiation			<0.001
High	Ref		
High-moderate and moderate	1.39	(0.44–4.39)	0.576
Moderate-poor and poor	2.31	(0.73–7.34)	0.154
Other	0.56	(0.06–5.61)	0.621
Nerve invasion	1.39	(0.79–2.45)	0.248
No (ref.)			
Yes			
Surgical margins	3.12	(1.44–6.80)	0.004
R0 (ref.)			
R1			
Surgical time	1.30	(1.03–1.66)	0.029
< 282.5 minutes (ref.)			
>= 282.5 minutes			
Intraoperative blood loss	1.22	(0.96–1.57)	0.102
< 450 (ref.)			
>= 450			
Postoperative complications	1.26	(1.10–1.57)	0.033
No (ref.)			
Yes			
Chemotherapy	0.61	(0.48–0.77)	<0.001
No (ref.)			
Yes			
NRS-2002 score	1.33	(1.06–1.67)	0.015
<3 (ref.)			
>=3			

We also used the Bonferroni correction to address the issue of multiple hypothesis testing, thereby reducing the probability of type I errors. The significance level was adjusted to 0.0028 (0.05/18 tests) in the univariate analysis. The multivariate analysis results showed that carbohydrate antigen 199 levels, NLR, lymph node metastasis, distant organ metastasis, tumor tissue differentiation, operative time, intraoperative blood loss, chemotherapy, and NRS-2002 score were independent prognostic factors in patients with pancreatic cancer after pancreaticoduodenectomy (P < 0.05) (**Supplementary Table 1**).

### The independent association between nutritional risk and survival

**Figure 1:**
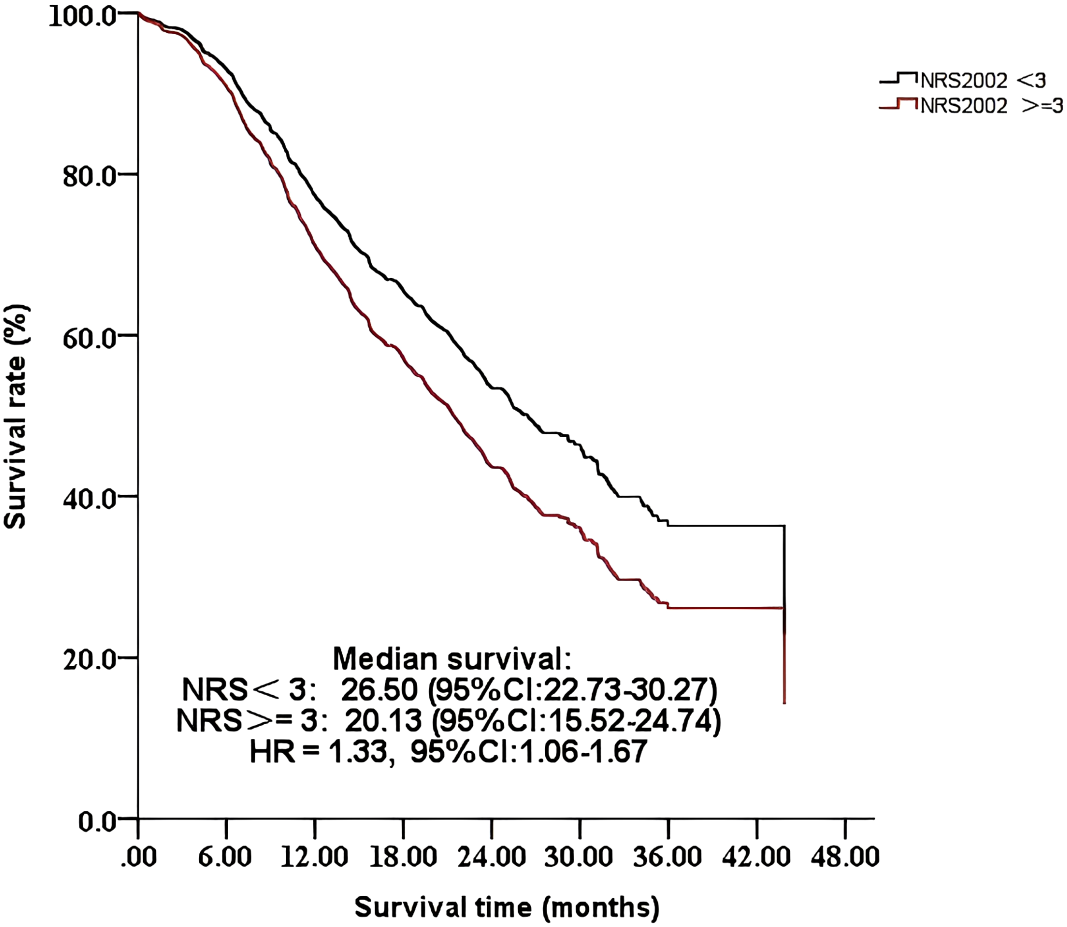
Comparison of the survival time of 656 patients with different NRS-2002 scores: <3 or >=3.

The results indicated that nutritional risk (NRS-2002 >=3) was associated with lower survival in an unadjusted model [hazard ratio (HR) = 1.47, 95% CI: 1.19–1.83]. This association was diminished after adjusting for different variables. After fully adjusting for age, education level, carbohydrate antigen 199 levels, NLR, tumor diameter, lymph node metastasis, distant organ metastasis, differentiation, nerve invasion, surgical margins, operative time, intraoperative blood loss, postoperative complications, and chemotherapy, this association remained (HR = 1.33, 95% CI: 1.06-1.67) (**Figure 1**). Detailed information is presented in **Table 4**.

**Table 4:** Cox regression analysis of the association between nutritional status and survival time.

Variable	Non-adjusted HR, 95% CI, *P*	Adjusted I HR, 95% CI, *P*	Adjusted II HR, 95% CI, *P*	Adjusted III HR, 95% CI, *P*
NRS-2002 score >=3 vs <3 (Ref.)	1.47, 1.19–1.83, <0.001	1.40, 1.13–1.75, 0.002	1.34, 1.08–1.67, 0.009	1.33, 1.06–1.67, 0.015

I: age, education level, carbohydrate antigen 199 levels, and neutrophil-lymphocyte ratio (NLR).

II: age, education level, carbohydrate antigen 199 levels, neutrophil-lymphocyte ratio, and lymph node metastasis.

III: age, education level, carbohydrate antigen 199 levels, NLR, tumor diameter, lymph node metastasis, distant organ metastasis, differentiation, nerve invasion, surgical margins, operative time, intraoperative blood loss, postoperative complications, and chemotherapy.

HR, hazard ratio; CI, confidence interval.

## Discussion

With the refinement of surgical techniques and improvements in neoadjuvant and adjuvant therapies and the application of early diagnostic techniques, the survival time of patients with pancreatic cancer has been prolonged. In this study, we found that the overall 1- and 3-year survival rates of the 656 patients with pancreatic cancer after pancreaticoduodenectomy were 72.7% and 34.4%, respectively, which were higher than the rates of 46.2% and 18.6% found in a previous study conducted in China (7). However, the reported data on survival remain lower than that from the JASPAC 01 (17) and PRODIGE-24 trials (18) in Japan and Canada, respectively. Thus, we still have a long way to go in improving the survival of patients with pancreatic cancer.

Similar to a previous study (19), our results confirmed that preoperative nutritional risk has a detrimental impact on survival in patients with pancreatic cancer who undergo a pancreaticoduodenectomy, and this relationship was stable. Conversely, Heckler et al. found that nutritional risk defined by NRS-2002 was not associated with worse survival in 116 patients with resected pancreatic ductal adenocarcinoma. The difference may be a result of different populations and sample sizes. Patients with pancreatic cancer commonly experience metabolic dysfunction, systemic inflammation, and unintentional body weight loss due to tumor-induced and treatment-associated changes in physiological function (20), which makes patients with pancreatic cancer more susceptible to nutritional risk. Nutritional risk is associated with a worse prognosis in patients with cancer (21). Pan et al. found that for cancer patients with nutritional risk, the relative risk of adverse events significantly increased compared with patients without nutritional risk (22). Moreover, nutritional risk decreases tolerance of curative cancer treatments, such as surgery and chemotherapy, leading to significant reductions in therapeutic effects (23). This adversely and severely affects cancer prognoses (24). Our results indicate that early screening for nutritional risk using the NRS-2002 and a corresponding intervention program for patients with pancreatic cancer before surgery are urgently required to improve the poor survival time. Furthermore, our survival curves show that the decline was gradual in the group without nutritional risk, while it was steeper in the group with nutritional risk. The curves appear to run parallel after approximately 18 months, indicating that the nutritional risk at baseline has a stronger influence on early rather than late mortality. Prior research has also demonstrated that the long-term survival prognosis for pancreatic cancer is more susceptible to the inherent characteristics of the tumor and the nature of the treatment (25).

Consistent with some previous reports (7–9, 19), we found that age, gender, and smoking habits had no effect on the prognosis of patients with pancreatic cancer, and higher carbohydrate antigen 199 levels, lymph node metastasis, distant organ metastasis, poor differentiation, resection margin status, postoperative complications, and absence of chemotherapy predict a poor prognosis in patients with pancreatic cancer. However, in contrast to some previous studies (7, 26), other previous studies have found that NLR, a commonly used indicator of systemic inflammation, with cutoff values ranging from 2 to 3.8, is a significant prognostic indicator for overall survival in patients undergoing pancreaticoduodenectomy (27). Similar to the previous reports, we also found that patients with NLR greater than 2.93 had significantly shorter survival than patients with NLR less than 2.93. Furthermore, nerve invasion was not associated with survival in patients after pancreaticoduodenectomy in this study. This result is different from the study of Sugimoto et al., which found that extrapancreatic nerve invasion was associated with a shorter disease-specific survival and recurrence-free survival after upfront surgery in patients with anatomically resectable pancreatic cancer (28). The difference may be due to the variation of nerve invasion diagnosis, which depends on personalized experience and on the internal protocol adopted for pathological sampling, together with its examination by experienced pancreatic pathologists (29). Future studies should focus on using standardized methods to assess nerve invasion and include larger patient cohorts to explore the interaction between nerve invasion and other prognostic factors.

Several limitations should be mentioned in our study. First, due to the limited cases with neoadjuvant chemotherapy, we analyzed the effect of chemotherapy on survival in pancreatic patients combined with adjuvant and neoadjuvant chemotherapy. We did not consider disease-free survival or cancer-specific survival in our analysis of the results, which are crucial indicators for evaluating postoperative prognosis in cancer patients. Additionally, lymphovascular invasion and vascular resection have been reported in previous studies to have a possible effect on survival after pancreatic cancer surgery, but we could not accurately obtain data on these two items in the medical record system, so they were not included as covariates in the analyses. Moreover, this is a single-center retrospective analysis, so larger multi-center studies are needed to confirm the results of this study.

## Conclusion

In conclusion, the prognosis of patients with pancreatic cancer who undergo pancreaticoduodenectomy is affected by many factors. Higher carbohydrate antigen 199 levels, lymph node metastasis, distant organ metastasis, poor differentiation, NLR, resection margin status, operation time, postoperative complications, and preoperative nutritional risk predict a poor prognosis in patients with pancreatic cancer after surgery. Our findings confirmed that this relationship between nutritional risk and poor survival is stable. Thus, in addition to early detection, timely surgery, and aggressive postoperative treatment, nursing staff should screen early for nutritional risk using the NRS-2002 in patients with pancreatic cancer at diagnosis and, in conjunction with their doctors, develop and implement a timely nutritional treatment plan for those at risk to improve the poor survival time.

## Data Availability

The raw data supporting the conclusions of this article will be made available by the authors, without undue reservation.
