# Supplementary material for: The association between nutritional risk and survival time among patients with pancreatic cancer following pancreaticoduodenectomy: a retrospective cohort study
Source: Front Oncol. 2025 Jul 1;15:1539215. doi: 10.3389/fonc.2025.1539215 (PMC12259460; doi:10.3389/fonc.2025.1539215)
Supplement: Supplementary file 1 [file Table1.docx]

Supplement table 1 multivariate analysis of prognostic factors for pancreatic cancer patient survival

| Variables | HR | *95%CI* | *P* |
| --- | --- | --- | --- |
| Age (years) | 1.22 | (0.96-1.56) | 0.108 |
| < 60 (ref.) |  |  |  |
| >=60 |  |  |  |
| Carbohydrate antigen 199 levels | 1.45 | (1.17-1.80) | 0.001 |
| <=305 (ref.) |  |  |  |
| >305 |  |  |  |
| Neutrophil-lymphocyte ratio | 1.42 | (1.14-1.77) | 0.002 |
| < 2.93 (ref.) |  |  |  |
| >= 2.93 |  |  |  |
| Lymph node metastasis | 1.67 | (1.34-2.07) | < 0.001 |
| No (ref.) |  |  |  |
| Yes |  |  |  |
| Distant organ metastasis | 2.11 | (1.40-3.20) | < 0.001 |
| No (ref.) |  |  |  |
| Yes |  |  |  |
| Differentiation |  |  | <0.001 |
| High | Ref |  |  |
| High-moderate and moderate | 1.40 | (0.44-4.41) | 0.567 |
| Moderate-poor and poor | 2.33 | (0.74-7.38) | 0.150 |
| Other | 0.61 | (0.06-6.13) | 0.687 |
| Surgical time (minutes) | 1.34 | (1.06-1.70) | 0.016 |
| < 282.5 minutes (ref.) |  |  |  |
| >= 282.5 minutes |  |  |  |
| Intraoperative blood loss (ml) | 1.32 | (1.04-1.68) | 0.023 |
| < 450 (ref.) |  |  |  |
| >= 450 |  |  |  |
| Chemotherapy | 0.62 | (0.49-0.78) | <0.001 |
| No (ref.) |  |  |  |
| Yes |  |  |  |
| NRS2002 value | 1.33 | (1.06-1.67) | 0.013 |
| <3 (ref.) |  |  |  |
| >=3 |  |  |  |
